# Cytotoxicity Enhancement in MCF-7 Breast Cancer Cells with Depolymerized Chitosan Delivery of α-Mangostin

**DOI:** 10.3390/polym14153139

**Published:** 2022-08-01

**Authors:** Yedi Herdiana, Nasrul Wathoni, Shaharum Shamsuddin, Muchtaridi Muchtaridi

**Affiliations:** 1 Department of Pharmaceutics and Pharmaceutical Technology, Faculty of Pharmacy, Universitas Padjadjaran, Sumedang 45363, Indonesia; nasrul@unpad.ac.id; 2 School of Health Sciences, Universiti Sains Malaysia, Kubang Kerian 16150, Malaysia; shaharum1@usm.my; 3 Nanobiotech Research Initiative, Institute for Research in Molecular Medicine (INFORMM), Universiti Sains Malaysia, Penang 11800, Malaysia; 4 USM-RIKEN Interdisciplinary Collaboration on Advanced Sciences (URICAS), Universiti Sains Malaysia, Penang 11800, Malaysia; 5 Department of Pharmaceutical Analysis and Medicinal Chemistry, Faculty of Pharmacy, Universitas Padjadjaran, Sumedang 45363, Indonesia

**Keywords:** anticancer, molecular weight, nanoformulation, nanoparticles, sustained release

## Abstract

The application of α-mangostin (AMG) in breast cancer research has wide intentions. Chitosan-based nanoparticles (CSNPs) have attractive prospects for developing anticancer drugs, especially in their high flexibility for modification to enhance their anticancer action. This research aimed to study the impact of depolymerized chitosan (CS) on the cytotoxicity enhancement of AMG in MCF-7 breast cancer cells. CSNPs effectivity depends on size, shape, crystallinity degree, and charge surface. Modifying CS molecular weight (MW) is expected to influence CSNPs’ characteristics, impacting size, shape, crystallinity degree, and charge surface. CSNPs are developed using the method of ionic gelation with sodium tripolyphosphate (TPP) as a crosslinker and spray pyrolysis procedure. Nanoparticles’ (NPs) sizes vary from 205.3 ± 81 nm to 450.9 ± 235 nm, ZP charges range from +10.56 mV to +51.56 mV, and entrapment efficiency from 85.35% to 90.45%. The morphology of NPs are all the same spherical forms. In vitro release studies confirmed that AMG–Chitosan–High Molecular Weight (AMG–CS–HMW) and AMG–Chitosan–Low Molecular Weight (AMG–CS–LMW) had a sustained-release system profile. MW has a great influence on surface, drug release, and cytotoxicity enhancement of AMG in CSNPs to MCF-7 cancer cells. The preparations AMG–CS–HMW and AMG–CS–LMW NPs considerably enhanced the cytotoxicity of MCF-7 cells with IC_50_ values of 5.90 ± 0.08 µg/mL and 4.90 ± 0.16 µg/mL, respectively, as compared with the non-nano particle formulation with an IC_50_ of 8.47 ± 0.29 µg/mL. These findings suggest that CSNPs can enhance the physicochemical characteristics and cytotoxicity of AMG in breast cancer treatment.

## 1. Introduction

Advances in biomaterials-based drug delivery systems (DDS) are urgently needed and have expanded available options for effective, efficient, cost-effective, and bio-compatible alternative medicine and warrant eradication of the resistant cancer cells [1]. Cancer is a life-threatening disease which is one of the greatest health challenges of mankind and requires a positive treatment plan [2]. The most frequently diagnosed cancer worldwide is breast cancer (2.26 million cases) [3,4,5], and it is the fifth leading cause of cancer death in women [6]. However, there are disadvantages of various therapies hindering the success of clinical treatment [2]. Surgery, chemotherapy, and radiotherapy have become the mainstay of cancer treatment. The problems that arise are metastasis, drug resistance, toxicity, and unwanted side effects [7], and the incidence of cancer relapse that results from remaining malignant cells and the presence of cancer stem cells is still frequently encountered [1].

However, the lingering challenges in cancer therapy persist, and a major snag has been the resistance of cancer cells towards synthetic drugs [8]. The ongoing quest for better and more efficient chemoprevention and chemotherapeutics is important. Phytochemicals have recently gained expanded interest as chemoprevention and chemotherapeutics [9,10,11]. Our previous study shows that AMG, as a natural medicine, is a potential chemopreventive and chemotherapeutic substance used for cancer treatment [12]. The antitumor activity of AMG can act at almost all major stages of tumor development, leading to cell cycle arrest and cellular apoptosis in several human cancer cell lines and has better selectivity, but the application of AMG is limited due to its hydrophobic properties, poor solubility, and stability in water. Therefore, AMG has low bioavailability and accumulation in target organs [12,13].

Polymeric NPs have great potential for hydrophobic drug delivery, increase drug solubility, prolonging drug residence time, and improving stability [14,15]. Polymeric NPs are among the most scientifically investigated and have more benefits, such as low manufacturing costs, harmonious design, less harmfulness [16], use as controlled release vehicles, the ability to shield drugs and other biologically active molecules from the environment, and the improvement of their bioavailability and the therapeutic index [17].

CSNPs in cancer therapy are expected to increase drug accumulation in tumors, accuracy of drug delivery to target sites, higher solubility of pharmaceutical properties, and lower systemic toxicity [7]. Among NPs, biopolymeric NPs such as CS are mainly used for cancer therapy goals because of their substantial advantages such as cost-effectiveness, ease of manipulation, biocompatibility, mucoadhesiveness, biodegradability, enhanced permeation, eco-friendly properties [18,19,20,21], stability, solubility, and pH-sensitive CS nanoparticles [8].

The physicochemical and biological characteristics of CSNPs are strongly influenced by their MW and surface charge [22,23,24,25]. The surface charge of the NPs impacts the accumulation of NPs at the target site, stability, cellular uptake, protein adsorption applications, and distribution throughout the body. The positive charge of CS has a significant interaction with a variety of substrates and cells in the body, especially tumor cells [23]. In nanomedicine, size also plays a significant influence in shaping its biological function. The distribution of particle size (PS) is determined by several variables, such as mixing of CS/TPP, the concentration of CS, degree of deacylation (DDA) and MW, ionic strength, and pH of the medium [23,26,27]. As a type of water-soluble CS, low MW¬–CS (CS¬–LMW) has shown outstanding advantages as a drug carrier, nontoxicity, biocompatibility, biodegradability, and ability to increase absorption [26]. CS has three different functional groups that can take part in various chemical and physical reactions [27]. The hydrophilicity of the polymer is due to the hydrophilic groups such as amine, hydroxyl, and carboxylate, which are distributed throughout the polymer structure [28].

Various techniques have been used to prepare CSNPs. Most of the major crosslinkers, surfactants, and certain grafting agents used in the preparation of CSNPs are not free from toxicity, which is important to account for long-term exposure. The ionic gelation method is based on ionic interactions between positively charged amino groups of CS and negatively charged polyanion groups. The most frequently used crosslinking agent is tripolyphosphate [29], so that the resulting NPs are more stable and strong [30].

**Scheme 1:**
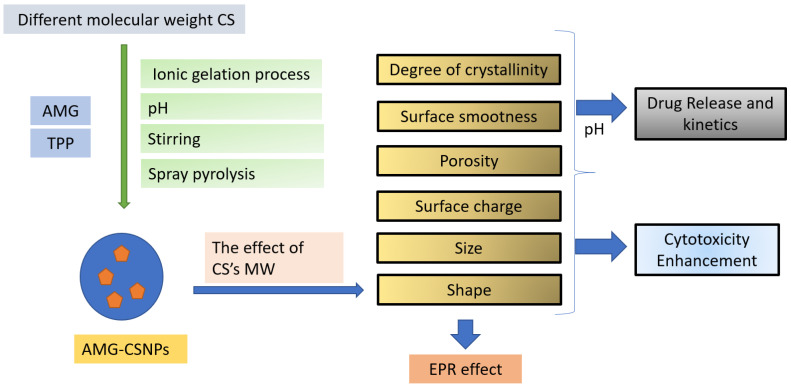
The general idea of the performed studies.

Modified CS can be made into multilayer composites by combining with polymers and other parts to change the properties of CS for certain biomedical applications, such as sodium alginate and chitosan [31]. CS can be encapsulated using kappa carrageenan [22] and Diethyl curcumin disuccinate [32]. CS–NPs were modified with either biotin or biotin and avidin [33]. CS coating can improve the physical stability of NPs [34] and overcome poor solubility in water and low selectivity against cancer cells. AMG in CS-kappa carrageenan can increase potential cytotoxic activity [22]. Modification of MW–CS affect the molecular weight, size, surface smoothness, porosity, shape, and charge of CSNPs, then change the kinetics of the effect of increasing permeability and retention (EPR), increasing AMG cytotoxicity (Scheme 1). This work contributes to the further development of nanosystems for treating breast cancer with AMG–CS.

## 2. Materials and Methods

### 2.1. Materials

AMG was purchased from Chengdu Biopurify Phytochemicals (Shincuan, China). CS–HMW with 300 kDa (82% DDA) was purchased from Interlab, Ltd. (Jakarta, Indonesia). Acetic anhydride was purchased from Interlab, Ltd. (Jakarta, Indonesia). All analytical grades of concentrated sodium hydroxide and absolute ethanol were provided from local suppliers. All chemicals were used as received without further purification. Zeta potential was performed using a Zetasizer SZ 100 Horiba (Kyoto, Japan). MCF-7 cancer cell was provided from the American Type Culture Collection (Manassas, VA, USA).

### 2.2. Preparation of Different Molecular Weights of Chitosan

Variation of CS molecular weights was obtained by the depolymerization of CS using sodium nitrite in acid media, dissolving 9.0 g of CS in each 450-mL (*v*/*v*) acetic acid (2%) solution and allowing for overnight at room temperature (RT). Two sets of separate solutions for CS were prepared. CS solutions were applied to 0.5% *w*/*w* NaNO_2_ at 500 rpm for 3.5 h at 30 °C, the solutions were slowly stirred and then neutralized with 5 M NaOH. The solutions were concentrated at 50–60 °C by rotary evaporator (Buchi Labortechnik, Flawil, Switzerland) until a volume of approximately 60 mL of concentrated solutions was obtained. The condensed solutions were poured into 100 mL methanol to remove the CS samples, washed with acetone, then dried overnight at RT. The CS samples were kept in the refrigerator before assessing the degree of deacetylation and the average molecular weight.

### 2.3. Characterization CS–LMW

#### 2.3.1. Characterization of CS DDA

Fourier-transform infrared spectrophotometer was used to characterize CS DDA (Model IR Prestige-21, Kyoto, Japan) and measured at 4000−400 cm^−1^. The DDAs of CS and LMW CS were calculated according to the following Equation (1) [35]:

DDA = 100 × (1 − (A_1655_/A_3450_)/1.33) (1)

which was derived for these absorbances.

#### 2.3.2. CS Molecular Weight Characterization

An acetic acid/sodium acetate buffer solvent was prepared by mixing equal volumes of 0.25 M acetic acid and 0.25 sodium acetate solutions. The pH of the buffer was measured and then readjusted to pH = 4.0 using sodium hydroxide solution. One gram of chitosan sample was dissolved in 125 mL of the buffer solvent system. The resulting solution (0.8 g/dL) was continuously stirred for 3–4 h until the chitosan had completely dissolved. The average molecular weight of chitosan was calculated by applying the Mark–Houwink’s Equation (2) [36].

(2) $$\left[\eta \right]=k\;Mv^{\alpha }\;\left(acetic\;acid\right)$$

where *k* = 1.64 × 10^−30^ × (DDA%)^14^, and α = −1.02 × 10^−2^ × (DDA%) + 1.82.

#### 2.3.3. Fourier-Transform Infrared Analysis

Fourier-transform infrared spectrophotometer was used to characterize CS–LMW (Model IR Prestige-21, Kyoto, Japan) and measured at 4000−400 cm^−1^ [30].

#### 2.3.4. X-ray Diffraction Analysis

X-ray powder diffraction (XRD) method (X-pert MPD diffractometer type, Rigaku International, Tokyo, Japan) was used to identify CS crystalline phase in the samples. The molecular arrangements of CS–LMW and the initial CS system were observed over the angular range (2θ) of 5–60° [37,38].

### 2.4. Preparation of CS NPs

A modified ionic gelation technique was used in synthesizing CSNPs. The formulas of the nanoparticles are shown in Table 1. CS was dissolved in an acetic acid (0.2 mg/mL) to obtain a CS concentration 0.5 mg/mL. At a pH of 4.7–4.8, the CS solution was agitated overnight at room temperature using a magnetic stirrer. After filtering the CS solution using a syringe filter (0.45 µm), it was warmed in a water bath at 60 °C for 10 min. TPP was dissolved in distilled water at various concentrations, filtered through a syringe filter (0.22 µm), and chilled to 2–4 °C in another beaker. Finally, five milliliters of TPP solution were added to ten milliliters of CS solution and mixed for ten minutes [39]. Spray pyrolysis equipment was used to obtain NPs [40]. This method was performed for the manufacture of CS–LMW NPs.

### 2.5. Characterization of CS NPs

#### 2.5.1. Particle Size (PS) and Zeta Potential (ZP)

The mean PS and ZP of the NPs were measured using a Zetasizer SZ 100 Horiba (Kyoto, Japan). All measurements were carried out in triplicate [41].

#### 2.5.2. Nanoparticles Morphology

The surface morphology characterization of nanoparticles was carried out using scanning electron microscopy (SEM). The nanoparticle powder is placed on a stub using adhesive on both sides. Then, the powder is made to be electrically conductive with a beam of thin platinum (coating) for 30 s at a pressure of 10 mA. The photo is taken at 10 kV with the desired magnification. SEM (Thermo Scientific, Braunschweig, Germany) and TEM (Thermo Scientific, Braunschweig, Germany) were used to study the morphologies of all CSNPs. Before analysis, the samples were coated in carbon film and examined under a microscope.

#### 2.5.3. Fourier-Transform Infrared Analysis

Procedure 2.3.3 for AMG, CS, TPP, and AMG–CS NPs.

#### 2.5.4. X-ray Diffraction Analysis

Procedure 2.3.4 for AMG–CS NPs.

#### 2.5.5. Encapsulation Efficiency and Drug-Loading Capacity

EE of AMG and DL of NPs was determined by UV-VIS spectroscopy. A total of 25 mg of sample NPs was dissolved in ethyl acetate and then was centrifuged (3000 rpm, 10 min). The absorption of supernatant was measured by a UV-visible spectrophotometer at 245 nm to determine free AMG [42]. The sediment was resuspended in ethanol to determine the encapsulated drug to find the total amount of AMG. A standard curve was obtained using the different concentrations (2–20 µg/mL) measured at 245 nm. The EE and DL of AMG present in NPs were calculated using the following Equations (3) and (4) [43,44]:

(3) $$\mathrm{Entrapment}\;\mathrm{efficiency}\;\left(\%\right)=\frac{\mathrm{mass}\;\mathrm{of}\;\mathrm{mangostin}\;\mathrm{present}\;\mathrm{in}\;\mathrm{nanoparticle}\;\left(\mathrm{mg}\right)\;}{\mathrm{mass}\;\mathrm{of}\;\mathrm{mangostin}\;\mathrm{used}\;\left(\mathrm{mg}\right)}\;\times 100\%$$

(4) $$\mathrm{Drug}\;\mathrm{loading}\;\left(\%\right)=\frac{\mathrm{mass}\;\mathrm{of}\;\mathrm{mangostin}\;\mathrm{present}\;\mathrm{in}\;\mathrm{nanoparticle}\;\left(\mathrm{mg}\right)\;}{\mathrm{total}\;\mathrm{mass}\;\mathrm{of}\;\mathrm{nanoparticle}\;\left(\mathrm{mg}\right)}\;\times 100\%$$

### 2.6. In Vitro Drug Release Study

The release activity of AMG from NPs was investigated at pH 4.0 (pH in endosomes or lysosomes), pH 6.0 (pH around the tumor), and pH 7.4 (pH of physiological blood) [45,46,47]. AMG–CS NPs were dispersed in PBS and transferred into a dialysis membrane tube (Ward Science, West Henrietta, NY USA, MW cut-off 14000 Da). The 20 mg NPs were immersed in 60 mL of PBS for the in vitro release test and incubated at 37 °C. Each sample of PBS was 5 mL in volume, and samples were obtained at intervals of 2, 4, 6, 8, 10, and 12 h. The AMG content was evaluated by UV-spectrophotometry [48,49]. The release profile was determined by graphing the cumulative AMG release from the matrix as a function of the time spent immersing in the PBS liquid.

### 2.7. In Vitro Cytotoxicity

The metabolism of the tetrazolium substratum MTT (3-[4,5-dimethylthiazol-2-yl]-2,5 diphenyl tetrazolium bromide) determined MCF-7 cell growth. A total of 10,000 MCF-7 cells/well were seeded onto a 96-well plate for 24 h. Subsequently, the medium was replaced with extracts at varying quantities and incubated for 12 and 24 h. The medium was withdrawn and incubated with MTT solution at 5 g/L in PBS, pH 7.4, for 4 h. There was no more cultivated media; 150 microliters DMSO was added. After dissolving the formazan precipitate, the plate. Was gently agitated. On average, 570 nm absorbance is used compared with 630. A dip in absorption indicated a loss in cell viability. The 50% median lethal concentration (LC_50_) was taken from the best-fit line obtained through linear regression analysis [50].

### 2.8. Statistical Analysis

All the measurements were made in triplicate, and the mean ± standard error of the mean was represented as all values. The findings were subjected to review by the *t*-test/t-student. If the *p*-value was 0.05, the findings were considered statistically significant.

## 3. Results and Discussions

### 3.1. Characterization of CS–LMW

#### 3.1.1. DDA and MW Characterization

The MW and DDA of the initial CS were determined and found to be 300 kDa. Furthermore, CS–LMW was prepared by adding NaNO_2_ solution.

The depolymerization reaction of CS–HMW to CS–LMW can be carried out enzymatically, physically, and chemically [51]. Industrially, chemical treatment for acid degradation is preferred because it is convenient, low-cost, fast, scalable, and produces reproducible CS–LMW mixtures [52,53]. The use of NaNO_2_ showed the best performance for CS degradation [39]. Variations in the amount of NaNO_2_ and reaction time obtained two samples of CS with Mw 20 kDa (Table 2). Samples prepared from the initial CS (Mw 300 kDa) were then used to compare the properties.

In this study, the Mark–Houwink equation was used to calculate the average MW [54]. The percentage of DDA is calculated from the FTIR spectrum, using this equation: DDA = 100 × (1-(A1655/A3450)/1.33). The CS–LMW was 20 kDa with a DDA of 75.0%. This CS was classified as CS–LMW (<50 kDa) [51]. CS–LMW with high DDA is expected to dissolve in a much wider pH range than CS–HMW. The DDA of CS influences the polymer’s biodegradability by describing the fraction of free primary amino groups. CS is mildly cytotoxic and non-biodegradable over 90% DDA but is destroyed by macrophages and neutrophils between 50% and 85% DDA. DDA and MW impact in vivo biodegradability. High MW degrades slowly in vivo, increasing its propensity to accumulate in tissues [51].

#### 3.1.2. Fourier-Transform Infrared Analysis

The CS–HMW and CS–LMW were characterized using a Fourier-transform infrared spectrophotometer (Shimadzu, Japan) and measured 4000−400 cm^−1^.

FTIR is an excellent method to identify chemical bond forms in a molecule by creating a spectrum of infrared absorption like a “molecular fingerprint“ [55]. FTIR spectroscopy has been shown to differentiate CS–HMW and CS–LMW (Table 3) [41]. 

**Figure 1:**
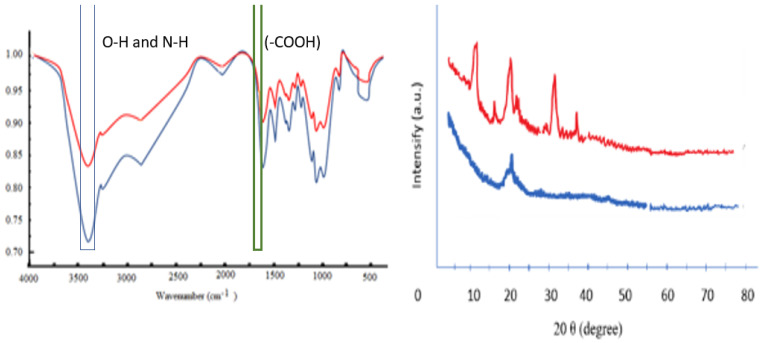
FTIR spectra and XRD spectra (1) CS–HMW (blue line); (2) CS–LMW (red line).

Graphs A (blue line) and B (red line) in Figure 1 show the differences in the IR spectra of CS–HMW and CS–LMW. For stretching, vibrations O-H and N-H are represented in the band of about 3422 cm^−1^. At 1599 cm^−1^, the absorption peak correlated with the binding vibration of the amido group. The remaining acetyl is due to a clear carboxyl band (−C = O) at 1654 cm^−1^. Bands in the range of 1157 cm^−1^ to 896 cm^−1^ belong to the specific −1.4 glycosidic bond absorption peak in CS. The properties described above were also found in the CS–LMW FTIR spectrum. The presence of a new absorption peak at 1720 cm^−1^ was observed in the CS–LMW spectrum, which was determined for the absorption of the carboxylate group (-COOH) (green box) to be the difference between the two [56]. The findings confirmed that cleavage of the (−1, 4) glucoside bond in macromolecules is the basic mechanism of acid defense during the CS amino group without contributing to the ring-opening oxidation of repeated glucosamine units [24,41].

#### 3.1.3. X-ray Diffraction Analysis

XRD results (Figure 1) of CS–LMW show an amorphous pattern. Four crystal reflections in the range 2 (5–80°) are seen on the original CS–LMW X-ray diffractogram, indexed as 14.50°, 20.1°, 31.5°, and 36.75°. The profile of the diffraction peak shows certain modifications. However, the CS–LMW power from the diffraction angle is reduced. CS is the first to be reduced to water-soluble molecules in the amorphous region, and the solubility of CS in water increases [57].

CS has very strong intramolecular and intermolecular hydrogen bonds, making it a crystalline or semicrystalline material with various allomorphs [57]. Intramolecular interactions between O3 and O5 atoms can occur through glycosidic bonds stabilized by acetyl groups, whereas intermolecular interactions can occur through glucosamine units between N2 and O6 atoms. The decrease in the degree of crystallinity of CS is due to depolymerization [58,59,60,61]. Hydrogen bonds are believed to play an important role in the crystal structure. Functional groups such as hydroxyl and amine CS are excellent hydrogen bond donors and acceptors [62]. The increase in the N-glucosamine sequence increases the amorphous part of the structure because the removal of the acetyl group promotes the destabilization of the hydrogen bonds (HO3···O5) between the acetamide groups. The deterioration begins predominantly in the amorphous area and then moves very slowly from the crystal zone’s border to its center [49,51,62,63]. The crystal structure is broken, and crystallinity decreases with deeper damage [41,63]. In ideal circumstances, characterization of initial CS and LMW-CS revealed no change in chemical structure, while crystallinity reduced after degradation [41].

The mechanical strength, swelling, hydrolytic, and biodegradation rates of CS depend on the crystallinity, which is determined by the nature of the monomer. When the polymer chains have a stereoregular structure, the linear polymers with high MW can be rearranged into crystallites. Amorphous regions separate the crystalline domains because the polymer never reaches 100% crystallinity and is therefore semicrystalline. Crystallinity has less impact on drug release [64]. The amorphous character and large surface area of these particles compared with commercial HMW CS alter the solubility [49].

### 3.2. Preparation of CS NPs

Through the ionic gelation process, stable NPs dispersions of CS samples with different MW were prepared. The most popular preparation of CS NPs is ionic gelation. The use of TPP is a CS crosslinker, which can be relied on to produce stable NPs in relatively large and safe quantities. The CS-TPP method can obtain NPs with PS around 100 nm and a positive charge [48]. Under acidic conditions, CS (pKa 6.3) is polycationic and displays NH_3_^+^ sites and Sodium tripolyphosphate (Na_5_P_3_O_10_) dissolved in dissociated water [65]. Optimal pH 4.0–6.5 forms electrostatic interactions of positively charged amino groups of protonated CS with trivalent tripolyphosphate anions. With this method, it is possible to control the properties of the NPs, the absence of side reactions, and the safety of the tripolyphosphate [66].

Since CS cross-linking is based on the availability of cationic sites and negatively charged species, it is thought that pH plays an important role in the cross-linking process. Only phosphate ions are present when the pH of the TPP is converted to acid, CS-TPP complex occurs optimally at lower pH [67]. Among these modifications, cross-linking impacts valuable properties, for instance, chemical stability, inherent mechanical strength, swelling capability, solubility, and drug release, modifying aspects of CS. Cross-linked CS derivatives (CL-CS) show a pH-adaptive swelling tendency with hindered solubility in their porous structure. The loaded drug can be released from CL-CS by swelling the CS matrix on the gastric acidic pH and drug diffusion through the pores in a sustained release manner [54]. Drug delivery from polymer systems is influenced by several factors: polymer molecular weight, degree of deacetylation and substitution of polysaccharides, nanoparticle size, and porous structure. Polysaccharide chain length and conformation affect the accessibility of CS functional groups by drug molecules, which is critical for establishing electrostatic interactions and subsequent incorporation of drug molecules into this system [49]. The spray pyrolysis method gives denser NPs due to air drying. In the case of low heating, the evaporation of the solution and the progress of crystallization are slow. As a result, solid (solid) particles are generated [68].

### 3.3. Characterization of CSNPs (HMW/LMW)

#### 3.3.1. TEM, SEM, Zeta Potential (ZP), Particle Size (PS), Entrapment Efficiency (EE), Drug Loading (DL), and Poly Disperse Index (PDI)

**Figure 2:**
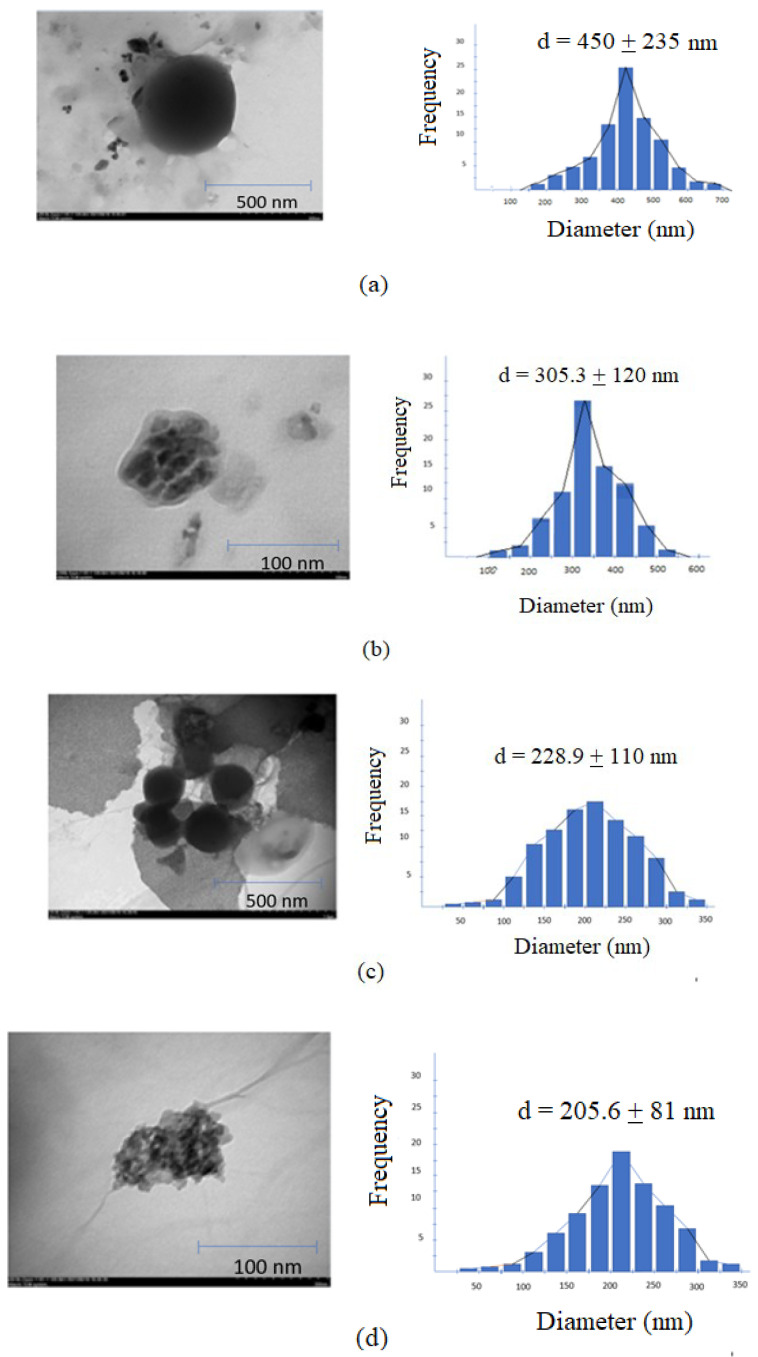
TEM images and size distribution analysis of (**a**) F1, (**b**) F2, (**c**) F3, (**d**) F4.

The TEM images of the AMG–CS NPs are seen in Figure 2. Morphological characterization of both AMG–CS NPs formulas formed spherical shapes. The PS, ZP, shape, EE, DL of NPs, and Poly Disperse Index (PDI) are shown in Table 4. 

**Figure 3:**
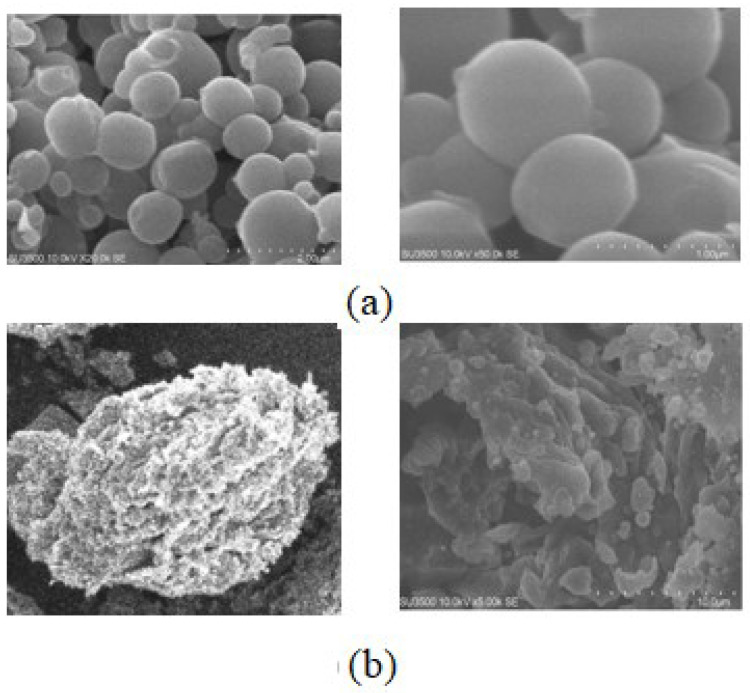
SEM images of (**a**) F1 (AMG–CS–HMW) and (**b**) F2 (AMG–CS–LMW).

Morphologically, CSNPs are identified by most studies as spherical particles [10,69]. This study showed that the shape of the NPs of CS–LMW and CS–HMW is spherical, as depicted in Figure 3. The spherical morphology of the particles was not affected by the increase in MW of CS [51]. Because of the complexation between oppositely charged species, CS undergoes ionic gelation and precipitates to form spherical particles [56]. All NPs formed in this study exhibited a spherical shape verified by SEM. The uptake of particles by cancer cells is determined by the intricate interactions between the physicochemical properties of the particles, such as shape, size, and surface functionalization [70]. The size varies from 5 to 200 nm depending on the length and shape of the polymer carrier. NP design (size, shape, surface, and stiffness) is critical for delivery efficacy due to several biophysical barriers, which prevent the circulation of NPs in the vascular flow and their accumulation at the tumor site [71].

The MW of CS affects the average PS and ZP. The increase in particle diameter is directly proportional to the increase in MW CS. The resulting AMG–CS–HMW NPs have a PS range of 228.3 ± 110 (nm), a ZP of 25.56 ± 3.4 (mV), and an EE of 88.36% ± 1.12 for AMG. AMG–CS–LMW NPs ranged in size from 205.3 ± 81 nm, with a ZP of 10.56 ± 2.2 (mV). For CS unloaded NPs, the diameters of prepared NPs were under 200 nm.

The NPs coated with CS¬–HMW usually showed a marginally higher mean diameter than CS–LMW (*p* > 0.05), possibly due to the higher MW, higher viscosity of the CS during the water process [72], and cross-links between TPP and CS. The rising PS with pH might be attributed to agglomeration as repulsive forces between CSNPs are reduced. The decrease in protonation of the NH_2_ groups on the CS contour led to reduced repulsive forces. The PS impacts drug dissolution. The Noyes–Whitney equation shows that more surface area or lower PS leads to quicker particle dissolution. In polymeric hydrogel particles, gradual drug dissolution followed by particle dispersion is expected. PS and surface area are important in material–biological interactions. The surface area of nanomaterials seems to expand exponentially with decreasing size, making them more reactive to themselves and their surroundings. Biological response and elimination are governed by PS and surface area [58,60,73]. Due to the EPR effect, as a “passive” target, such ranged NPs can aggregate more accurately in the tumor. The NPs increase in size marginally after the addition of the linker but retain the comparable ZP due to the negative charge of the NPs.

At pH 4.5, AMG–CS–HMW NPs had a higher mean ZP than AMG–CS–LMW NPs. This is because AMG–CS–HMW includes more positively charged NH_3_^+^ groups on the CS chain, which increases the surface charge of CSNPs. Moreover, during crosslinking, TPP ions were neutralized by the positively charged NH_3_^+^ groups of CS–HMW, which outnumbered TPP phosphoric groups. However, AMG–CS–LMW has fewer NH_3_^+^ groups to offset TPP’s negatively charged phosphoric groups. MW and CS types showed no significant influence on NP surface charge at pH levels other than 4 and 6.

NP sizes increased when CS MW fell because of the external phase’s viscosity. Less CS MW means less viscosity and hence smaller NPs. MW raised ZP. To understand this, consider the relationship between strand length and surface charge density. The amount of amine groups per strand decreases with strand length. Because amine groups must have a positive charge, the ZP falls with molecular weight. The PDI was higher in all NPs produced with low MW CS than in high MW CS [10,41,51,57,59]. The presence of free amino groups raised the surface charges and ZP of the NPs, strengthening the electrostatic contacts between the NPs and the drug, maintaining the spherical shape of the beads, and decreasing their size.

TEM images gain additional details, such as particle aggregation and agglomeration. Abdullah et al. reported TEM images of CNPs having a uniform spherical solid structure and having a nearly uniform particle size distribution. However, crosslinked CNPs (5%) tend to be low-aggregate [73]. In our case, it is possible that there are differences in ionic strength that affect the number of cross-links in the amino functional group of CS and differences in active substances. These two things are interesting to study further. The 3D SEM images of the CNPs show good dispersion of the nanoparticles to form a larger open surface area, making the CNPs particularly suitable for adsorption. The 2D TEM images unquestionably show that the CNPs exhibit highly porous surfaces due to their low agglomeration attributes. These porous CNPs and deagglomeration have been considered as key phenomena for the synthesis of novel CNPs, thereby maximizing their usefulness as nanomaterials in biomedical and agricultural applications, where their porous nature can effectively absorb harmful chemicals and antagonize pathogens, in our case useful in the release of active substances and enhancement of cytotoxicity [73,74]. Agglomeration and adsorption phenomena of CS NPs were observed by Mikušová et al. to reduce agglomeration; they physically fused CS NPs with fine lactose-PEG3000 microparticles, Lac/PEG3000 MPs (~5 µm), to reduce their agglomeration via the surface adsorption phenomenon [40]. The addition of microparticles in increasing cytotoxicity needs to be studied further.

#### 3.3.2. FTIR Analysis

**Figure 4:**
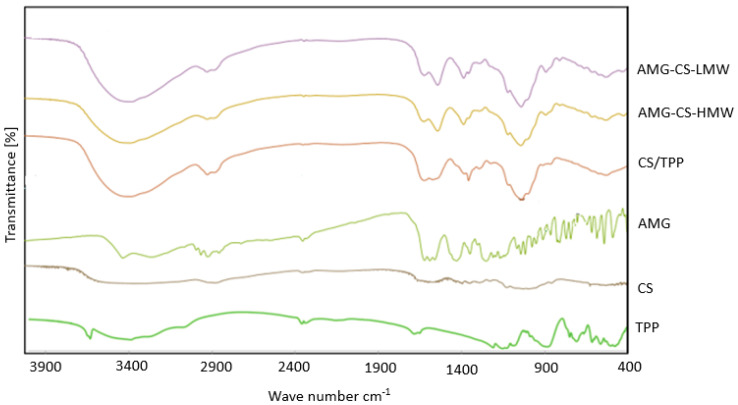
FTIR spectra of AMG, TPP, CS, AMG–CS Nps (HMW), and AMG–CS Nps (LMW).

By using FTIR to assess AMG–CS interactions, the ability of the ionic gelation mechanism to form AMG–CS–NPs was evaluated (Figure 4). Hydrogen-bonded O-H stretching vibration is due to the high and broad peak in the CS spectrum in the 3500–3300 region. In the same area, the peaks of N-H stretching from primary amine and amide form II are superimposed. The C-O-C asymmetric stretch peak is located at about 1150 cm^−1^, and the 1317 cm^−1^ peak belongs to the amine type I C-N stretching vibration. The tip of the peak of 3438 cm^−1^ has a shift to 3320 cm^−1^ in CS-TPP NPs and becomes wider with increased relative intensity, indicating an increase in hydrogen bonding. The amine I N-H bending vibration peaks at 1600 cm^−1^, and amide II carbonyl stretch at 1650 cm^−1^ changed to 1540 cm^−1^ and 1630 cm^−1^, respectively, in NPs. At 1170 cm^−1^, the crosslinked CS also shows a P = O peak. The linkage between phosphoric and ammonium ions has been attributed to these effects. Thus, we infer that the TPP tripolyphosphoric groups are related to the CS ammonium groups. In CSNPs, the inter-and intramolecular activities are improved [30,75].

#### 3.3.3. XRD Analysis

**Figure 5:**
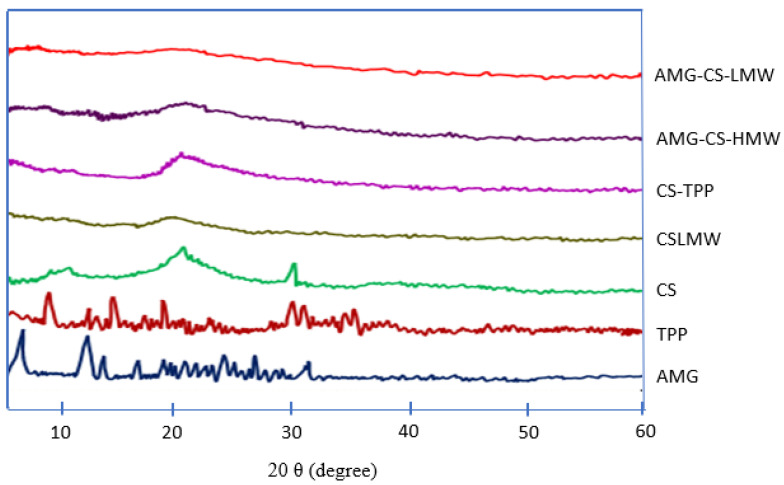
XRD spectra of AMG, TPP, CS, AMG–CS HMW, and AMG–CS LMW.

Figure 5 illustrates the XRD analysis findings. The XRD analysis of AMG–CS–LMW NPs demonstrated an amorphous pattern. AMG and TPP show crystalline patterns from diffractogram data with peaks from 2θ angles of 14–31.5°, and amorphous patterns are shown. The dissolution and solubility enhancement mechanism proposed in this study is “spring and parachute”, where amorphous drugs are often rapidly released upon dissolution, and the subsequent drop of supersaturation is caused by drug crystallization [76,77]. Water molecules can pass across amorphous areas, which are permeable. The monomers’ composition regulates the crystallinity and affects flexibility, swelling, solubility, and degradation rates. When low MW polymers are used, A high crystalline degree leads to slower drug release conditions. In high MW, the effect on drug release is reduced [23].

### 3.4. In Vitro Studies

#### 3.4.1. In Vitro Release

**Figure 6:**
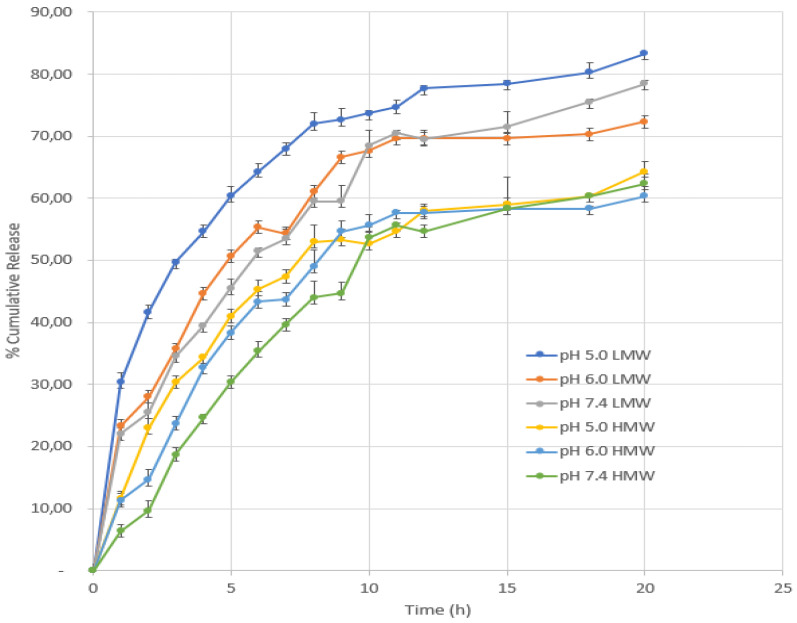
In vitro drug release profiles from CSNPs.

The need for effective medical therapy prompted the idea of controlled medication release. Targeted controlled release technologies outperform traditional medication delivery approaches [65]. In vitro studies in PBS were used to assess the AMG release pattern from NPs. In vitro release assays are required to predict if these systems would sustain a sufficient quantity of medication for the desired period of time AMG–CS NPs were tested for 20 h in vitro [78]. Drugs showed sustained release behavior for 12 h and then improved total drug release over 20 h (Figure 6). Due to the NPs’ small size, the formulation displayed an initial burst release. The true surface area grew as particle diameter dropped and distance to drug surface decreased. Due to their lower PS, AMG–CS–LMW NPs released quicker. The second is the HMW NPs’ resilience. The number of amino groups that undergo ionic interaction with TPP rises with CS molecular weight, increasing cross-linking density. Increased crosslinking enhances structural hardness and decreases active substance release. As shown, polymer concentration lowered active substance release. The density of TPP cross-linking rises with CS quantity, leading to more intensive microsphere structure development, and active drug release is reduced [58].

As seen in Figure 6, drug release was affected by pH. Protonation happened at a lower pH, releasing active substances into the medium. The positive surface of CSNPs at lower pH also reduced the drug–CSNP electrostatic interaction and promoted drug release. These data indicate the new DDS’s pH-triggered opioid releasing action. Most of the drug is expected to remain in the carrier for a long time (pH 7.4), reducing negative effects on normal tissues. However, once drug-loaded tumor cell CSNPs are endocytosed, the more rapid release may occur at lower local pH, at the tumor site, or within tumor cell endosomes and lysosomes, increasing the efficacy of cancer therapy. The pH of lysosomes, which destroy foreign particles and allow fast drug release inside cells, is 4.0–5.0 [78].

The CS–LMW significantly increased the AMG release, compared with the CS–HMW in CSNPs. To understand the kinetic release of AMG from the CSNPs in the first 24 h, we performed an analysis using Higuchi’s model [79,80]. The correlation coefficients (r) of the CSNPs (Table 5) suggests that the release type of AMG from the CSNPs was matrixtype based on a Fickian diffusion. Previously, we reported that the CS–LMW has a porous structure in the lack of NH_3_^+^. Therefore, the porous structure in CS–LMW may induce water uptake and promote the release of the AMG. Furthermore, the slope of the CS–LMW is higher than the CS–HMW, indicating the enhancement of AMG release rate.

#### 3.4.2. In Vitro Cytotoxicity

**Figure 7:**
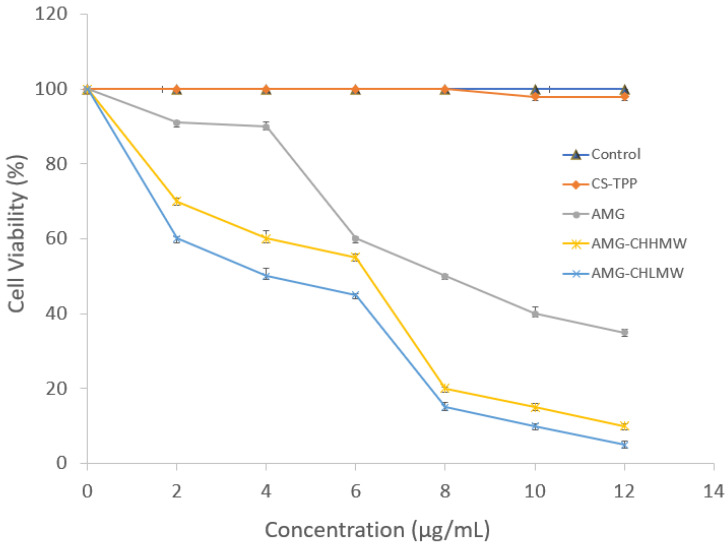
In vitro cytotoxicity of AMG, CS-TPP, AMG–CS–HMW NPs, and AMG–CS–LMW NPs MCF-7 cells based on the MTT assay.

The cytotoxicity of CS-TPP, AMG, AMG-CHLMW, and AMG-CHHMW NPs was determined by incubating with MCF-7 cells for 24 h (Figure 7). CS-TPP had no cytotoxic impact on cells. The cytotoxicity of AMG, AMG–CS, and AMG–CS LMW NPs was significantly different (Figure 7). AMG, AMG–CS–LMW, and AMG–CS–HMW had IC_50_ values of 8.47 ± 0.29, 5.90 ± 0.08, and 4.90 ± 0.16 g/mL respectively.

Cancer cells used in the MTT test have MCF-7 cells because of their sensitivity to estrogen receptors (ER) expression, so MCF-7 is good for studying hormone responses. MCF-7 cells have morphological-like epithelial and monolayer forming dome structures due to fluid accumulation between the culture disk and the monolayer of cells. Cytotoxicity assay was carried out using the MTT assay method (Figure 7). The redox reaction in cells is the basis of this approach. The succinate dehydrogenase enzyme present in live cells reduces MTT to formazan salt. After 4 h, a stopper reagent was introduced. The stopper reagent lyses the cell membrane and dissolves the formazan salt. The formazan salts produced were measured using a spectrophotometer. The greater the absorbance, the more viable cells [81] because of CS polymer’s mechanism as drug delivery has a very high amine group, which increases the CS affinity through electrostatic interactions to the cancer cell membrane.

The size of CSNPs is controlled by the MW and conformation of polymer CS. The smaller the NPs, the simpler it is to penetrate the cancer cell membrane, increasing the concentration of AMG in cancer cells. The cytotoxicity of AM-CS NPs formulations increased with increased concentration in cancer cells. Notable is the rise in vascular permeability from 200 to 780 nm in the developing solid tumor. They are tiny enough to pass via these pores from the blood to the interstitial tumor space. The EPR effect transports the pH-sensitive polymer micelles to the tumor site, where they are intracellularized by endosomes (pH 5.5–6.0) or lysosomes (pH 5.0) [82].

Large-scale studies of NPs in cancer treatment have shown that they may improve poorly soluble chemotherapeutic drug solubility, lengthen circulation duration in vivo, and increase accumulation in tumor locations [38,83].

The IC_50_ of NPs decreased from 8.47 ± 0.29 to 4.90 ± 0.16 µg/mL, indicating that CS MW affects anticancer cytotoxicity. The formulation of CSNPs with LMW significantly (*p* < 0.05) increased the cytotoxicity of αM against MCF-7 cells.

The CS MW (300 KDa and 20 KDa) of NPs altered their pharmacological capabilities. For example, CS MW and water viscosity impacted NP size. Using lower MW CS might minimize PS. Moreover, CS–LMW has increased water solubility, contributing to smaller particles and shorter polymer chains. The polysaccharide coating is evident by the positive ZP of all formulations [84].

## 4. Conclusions

Many approaches have been taken to improve the properties and expand the application window of CSNPs, including modifying the MW of CS. The properties of CSNPs such as the MW of CS affects size, charge, and release pattern of drugs and cytotoxicity. This research indicate that modified MW of CS may enhance AMG compounds’ physicochemical characteristics and cytotoxicity, so AMG–CS NPs can be the potential breast cancer treatment agents. Additional research is needed to clarify how various MWs of CS in CSNPs act on other breast cancer cell lines and normal cell lines and their feasibility for clinical application. This study still requires confirmation in vivo and selectivity tests on normal cells.

## Figures and Tables

**Table 1:** AMG–CS–NPs formulation.

Formulation	F1	F2	F3	F4
AMG (mg)	20	20	20	20
CS–HMW (mg)	200		100	
CS–LMW (mg)		200		100
Sodium tripolyphosphate (mg)	50	50	50	50

**Table 2:** DDA and Mw Value of CS–HMW and CS–LMW.

CS	NaNO_2_ (mg/mL)	DDA	M_w_ (kDa)
CS–HMW	0	80.0	300
CS–LMW	9	75.0	20

**Table 3:** FTIR wavenumber and corresponding functional groups.

Material	Result	Literature	Functional Groups Reference
CS–HMW	350,954	347,868	O–H stretch dan N–H stretch
	289,424	292,413	C–H stretch
	165,302	165,688	C = O
	1597	157,105	N–H bend [38]
	141,963	142,253	C–H bend
	137,816	137,816	C–N
	115,731	115,731	C–O–C stretch
	1079	102,518	C–O
CS–LMW	350,954	347,868	O–H stretch dan N–H stretch
	289,424	292,413	C–H stretch
	165,302	165,688	C = O
	1597	157,105	N–H bend [38]
	141,963	142,253	C–H bend
	137,816	137,816	C–N
	115,731	115,731	C–O–C stretch
	1079	102,518	C–O

**Table 4:** The mean EE and DL of the NPs.

Formula	PS (nm)	ZP (mV)	Shape	Entrapment Efficiency (%)	Drug Loading (%)	PDI
F1	450.9 ± 235	+51.56 ± 2.4	Spherical	90.45 ± 1.20	6.93 ± 0.87	0.487 ± 0.04
F2	305.3 ± 120	+15.69 ± 2.3	Spherical	89.35 ± 1.10	6.51 ± 0.54	0.394 ± 0.05
F3	228.3 ± 110	+25.56 ± 3.4	Spherical	88.36 ± 1.12	7.10 ± 1.70	0.435 ± 0.04
F4	205.3 ± 81	+10.56 ± 2.2	Spherical	85.35 ± 1.74	6.12 ± 0.86	0.375 ± 0.02

**Table 5:** Higuchi regression parameter of release percentage of drugs versus square root of time curves for release of AMG from CSNPs in PBS in 24 h.

Parameter	pH 7		pH 6		pH 4	
	CS–HMW	CS–LMW	CS–HMW	CS–LMW	CS–HMW	CS–LMW
Slope (%h^−0.5^)	23.33 ± 0.21	23.79 ± 0.72	23.40 ± 0.23	23.65 ± 0.21	20.80 ± 0.20	21.02 ± 0.21
Correlation coefficient (r)	0.98 ± 0.02	0.98 ± 0.01	0.95 ± 0.01	0.96 ± 0.01	0.96 ± 0.01	0.96 ± 0.02

## Data Availability

Samples of the data are provided by the corresponding author on request.
